# m5C Methylation of mRNA: Still More Questions Than Answers

**DOI:** 10.3390/cells15151336

**Published:** 2026-07-25

**Authors:** Elena S. Babaylova, Elizaveta A. Zolotenkova, Alexey A. Malygin

**Affiliations:** Knorre Institute of Chemical Biology and Fundamental Medicine, Siberian Branch of the Russian Academy of Sciences, Novosibirsk 630090, Russia; babaylova@1bio.ru (E.S.B.); zolotenkova@1bio.ru (E.A.Z.)

**Keywords:** m5C RNA methylation, mRNA, NSUN2, NSUN6, ALYREF, YBM1, gene expression, neurodegenerative diseases

## Abstract

mRNA plays a pivotal role in cellular processes of genetic information transfer, and post-transcriptional modifications of its nucleotides enable regulation of these processes with each particular mRNA. m5C methylation is a specific RNA modification that is rather common in tRNA, rRNA, lncRNA and other types of non-coding RNAs, whereas in mRNA it is found not very often. However, the functioning of m5C-methylated mRNAs differs from the non-methylated ones quite dramatically. Of the eight m5C RNA methyltransferases in humans, only two, NSUN2 and NSUN6, were found to be capable of modifying the main portion of cellular mRNAs. A deficiency of NSUN2 and NSUN6 causes global changes in the transcriptome and translatome, and a corruption of the *NSUN2* gene in humans is associated with neurodegenerative diseases and intellectual disability. Despite intensive research of m5C mRNA methylation in recent years, many aspects of this phenomenon and its significance remain problematic and far from understood. In this review, we discuss the available information on mRNA methylome biogenesis, methods for its study and analysis, effects of m5C modification on mRNA life, and place it in the general context of cell biology, attempting to draw the biological relevance of m5C mRNA methylation. We highlight the main inconsistencies and difficulties that arise, analyze their possible causes, and propose potential directions for further research that could clarify the controversial issues and provide a link between the NSUN2 deficiency and neurodegenerative diseases.

## 1. Introduction

Observed in the past years, a remarkable increase in the number of studies on m5C mRNA methylation is undoubtedly explained by the central role of mRNA in cellular pathways of the transfer of genetic information and, consequently, in the mechanisms of regulation of gene expression. It is stated, and there is much evidence, that m5C methylation of mRNA affects various aspects of its life, including nuclear-cytoplasmic export, stability, translational efficiency, subcellular localization, etc. It is clear that all of these processes are closely interconnected, and a change in the behavior of mRNA in one of them will inevitably affect its state in another, and it is often impossible to determine exactly what was the primary cause. Although numerous detailed reviews describing progress in studying m5C methylation of both RNA in general and mRNA in particular have been published recently (for example, [1,2,3,4,5,6]), much remains unknown and even contradictory when discussing m5C methylation of mRNA. Given all of the above, it is worth taking a closer look at the existing knowledge on this topic to understand the current state of the art and future prospects in this field.

## 2. Proteins Participating in the Biogenesis of mRNA m5C-Methylome

m5C is one of the most common modifications in various small and non-coding RNAs (such as tRNA, rRNA, vtRNA, snoRNA, lncRNA, etc.), i.e., in those RNAs whose spatial structure and stability are crucial for their processing or specific functioning. Therefore, it can be assumed that m5C methylation plays an important role in the processing and stabilization of the structure of these RNAs. This RNA modification is performed by methyltransferases of the NOL1/NOP2/SUN domain family (NSUN1-7) and the DNA methyltransferase homolog TRDMT1, which use S-adenosyl-L-methionine (SAM) as a methyl group donor, similar to DNA-specific methyltransferases. Notably, although NSUN7 belongs to the NSUN family of methyltransferases, recent studies show that it is unable to methylate RNA substrates [7].

**Figure 1:**
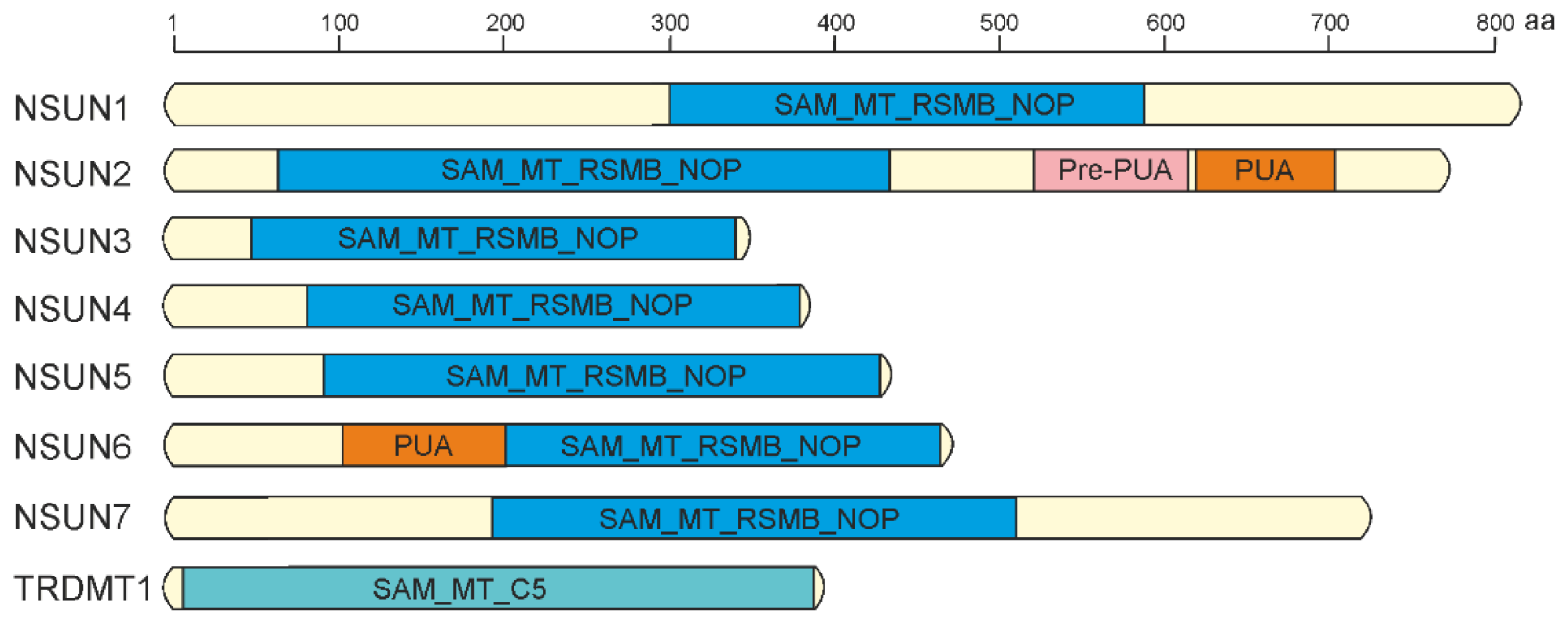
Domain structure of m5C RNA methyltransferases. Protein amino acid sequence lengths are presented on a uniform scale. Domain names (SAM_MT_RSMB_NOP—catalytic domain, pre-PUA and PUA—RNA-binding domains) and their boundaries correspond to those in the protein sequence classification resource InterPro [12].

All methyltransferases of the NSUN family, although differing substantially in size, share the SAM-dependent MTase RsmB/NOP-type domain, which contains a characteristic Rossmann-fold catalytic core (Figure 1). Methyltransferase TRDMT1 has a SAM MT C5 domain that performs the same function. In addition, NSUN2 and NSUN6 have a PUA domain, which is found in many RNA-binding proteins in all domains of life [8], and which may provide these methyltransferases with an additional RNA-binding site. It should be noted that although all RNA methyltransferases have specificity for different, unique RNA substrates, it is NSUN2 and NSUN6 that are additionally capable of methylating mRNAs [9,10,11] (a small portion of mRNAs may also be methylated by NSUN5 [11]).

**Figure 2:**
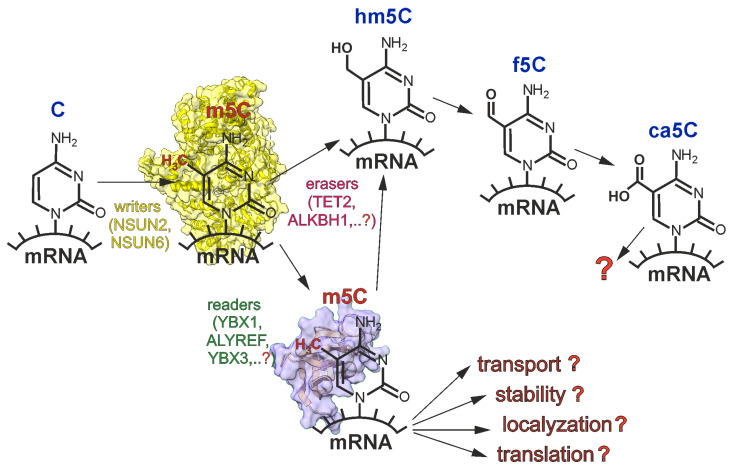
Schematic illustration of the mRNA m5C methylation pathway and subsequent processes involving the participating proteins.

Similar to proteins involved in the methylation of cytosine residues, m5C sites recognition, and removal of this mark in DNA, proteins participating in analogous processes in RNA are also termed “writers”, “readers”, and “erasers”. The writers are methyltransferases themselves; the following are typical RNA-binding proteins that prefer RNA regions containing m5C residues, and the latter are enzymes that stepwise oxidize and remove the methyl group from the cytosine residue. A general scheme of m5C mRNA methylation is shown in Figure 2. Below, we discuss various aspects of m5C mRNA methylation in more detail and highlight challenges in interpreting the results obtained in studying this process.

## 3. Identification of the m5C Site Positions in mRNA

A number of strategies using high-throughput sequencing have been proposed to determine the positions of m5C modifications in mRNA. The earliest method applied for this purpose was the bisulfite conversion assay (Bis-seq), which was originally used to detect DNA methylation and was later adapted to determine the position of m5C in RNA [9,13,14]. This method is based on the ability of the C residue, unlike m5C, to undergo deamination in a reaction with bisulfite to form a U residue. In high-throughput RNA sequencing (RNA-seq), those positions in mRNA where C-U conversion does not occur are considered the m5C positions. However, unlike DNA, free mRNA often has a complex secondary and tertiary structure, which can shield certain regions of the molecule from the action of bisulfite, thus producing false-positive signals in this method.

The next method, RIP-seq (RNA immunoprecipitation followed by sequencing), is based on the immunoprecipitation of mRNA fragmented into approximately 100-nucleotide fragments using antibodies specific to m5C and their subsequent high-throughput sequencing [15]. This method allows the direct detection of mRNA regions containing m5C residues but cannot precisely determine their location and also relies heavily on antibody specificity.

Before an enzyme can methylate a cytosine residue, it has to bind to the mRNA at the recognition site. In this logic, approaches based on the identification of NSUN enzyme binding sites on mRNA using chemical cross-linking appear productive. These include two methods: Aza-IP-seq (RNA immunoprecipitation with 5-azacytidine) [16] and miCLIP-seq (methylation individual-nucleotide resolution cross-linking and immunoprecipitation) [10], which utilize different variants of cross-linking of the enzyme to the cytosine residue located in its catalytic center. In the former case, a cytidine analog, 5-azacytidine, is used for this purpose. Cross-linked to the enzyme, RNA is isolated using enzyme-specific antibodies, and the cross-linked residue is identified by RNA-seq. In the second case, a mutant form of NSUN2, NSUN2(C271A), is used for cross-linking, which stabilizes the covalent bond between protein and RNA in the catalytic intermediate, and the cross-linked residue is also identified by RNA-seq. Thus, neither of these methods identifies the m5C sites in mRNA directly but instead indicates their potential location.

In addition to the application of cross-linking methods with a nucleotide residue in the active site of the enzyme, RNA-protein cross-linking methods can be used directly to determine contacts of the enzyme with mRNA in binding sites. One such method is the well-known PAR-CLIP (photoactivatable-ribonucleoside-enhanced CLIP) method [17], which utilizes photochemical cross-linking of a protein with 4-thiouridine-containing RNAs directly in the cell, followed by immunoprecipitation and identification of cross-linking sites and regions using RNA-seq. For example, the PAR-CLIP method was used for transcriptome-wide determination of NSUN2 binding sites with mRNA [18]. It is clear that this method allows identification only of those sites of protein contact on mRNA that contain a uridine residue replaced by 4-thiouridine; that is, the protein contacts with sites that do not contain uridine cannot be found in principle.

Recently, a number of other indirect methods for determining the positions of m5C in mRNA based on their recognition by “readers” and “erasers” have also been proposed, for example, m5C-TAC-seq (ten-eleven translocation (TET) enzyme-assisted chemical labeling sequencing) [11,19], TAWO-seq (TET-assisted peroxotungstate oxidation sequencing) [20], and DRAM (deaminase and reader protein-assisted RNA methylation analysis) [21]. These methods have expanded the tools available for m5C site mapping.

All the above methods have made it possible to detect thousands of m5C sites in mRNA from various human cells, depending on their type, experimental conditions, and other parameters. It has been shown that the majority of m5C methylation in mRNA is carried out by the methyltransferase NSUN2 [11]. However, it should be noted that all methods do not provide unambiguous results for m5C site mapping and may contain both false-negative and false-positive results. A comparison of results obtained using Aza-IP, Bis-Seq, and miCLIP revealed that the overlap of methylation sites detected by these methods is very low [22]. This means that data obtained using these and other existing methods should be treated with great caution. This also appears to apply to databases (e.g., “m5C Atlas” [23]) created to systematize available information on the location of m5C methylation sites in mRNA, which many researchers use in their work.

## 4. mRNA m5C Writers

The main events of m5C RNA methylation occur in the cell nucleus, where numerous tRNAs, snRNAs, vtRNAs, and rRNAs are methylated, and m5C methylation itself is partly a stage of their processing. Therefore, it is logical that many m5C methyltransferases are localized primarily in the nucleus. However, some of them are found in other cellular compartments. In particular, NSUN3 and NSUN4, which modify mitochondrial RNAs, are localized in the mitochondria [24,25], and NSUN6 is localized in the cytoplasm [26]. Methyltransferase TRDMT1 is both a cytoplasmic and a nuclear protein [27]. NSUN2, in addition to the nucleus, can also be found in mitochondria [28] and in cytoplasmic vesicles in the G2 phase of the cell cycle [29].

The majority of m5C modifications are introduced into mRNA by two enzymes, NSUN2 (to a greater extent) and NSUN6 [30]. Both of these proteins are capable of modifying mRNA in addition to their primary RNA substrates, specific tRNAs (for both enzymes) and vtRNAs (for NSUN2). Thus, based on the subcellular localization of NSUN2 and NSUN6, it can be expected that m5C methylation of mRNA can occur both in the cell nucleus, possibly even co-transcriptionally, and in the cytoplasm.

Methyltransferase NSUN5, whose target is 28S rRNA in the nucleolus, was also found to be capable of modifying mRNA [30], indicating an alternative subcellular localization in addition to the nucleolus. Some authors have also received indications that NSUN7, which plays an important role in spermatogenesis and is localized in the cytoplasm within vesicular structures in certain cancer cells [31,32], can also mediate mRNA methylation [33,34]. However, NSUN7 is not a methyltransferase per se, as it lacks catalytic activity due to its inability to bind SAM [7].

**Figure 3:**
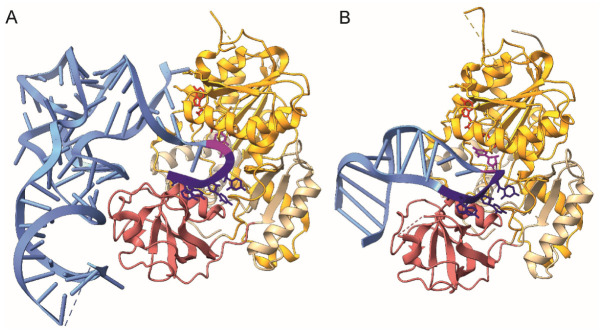
Crystal structures of human NSUN6 bound to its RNA substrates illustrating the common features and differences in the recognition of tRNA and mRNA by the enzyme. (**A**) Complex of NSUN6 with tRNA^Cys^ and a SAM analog (PDB: 5WWR [35]). (**B**) Complex of NSUN6 with the NECTIN-2 mRNA 3′-UTR fragment and a SAM analog (PDB: 9IMB [37]). Color code: RNA, blue; methylated C-residue, magenta; the UCCA site, deep blue; NSUN6, wheat; its catalytic core, gold; PUA domain, coral red; a SAM analog, red. It is seen that the PUA domain is involved in the binding of RNA structures in different ways, ensuring the positioning of the C residue in a conservative context into the active site of the enzyme. The figure has been prepared with the use of UCSF ChimeraX [38].

The spatial structure of m5C-methyltransferase in complex with its substrate, revealing the catalytic mechanism of its action, has been determined for NSUN6 [35,36,37] (Figure 3). It was found that, upon binding to tRNA^Cys^, the protein alters the conformation of the 3’ terminus of tRNA, allowing the enzyme’s PUA domain to bind the CCA-end and the D-stem region of tRNA [35]. Another part of the protein, the RRM of the MTase domain, discriminates residue U73 so that the methylation target C72 is located in a Rossman-fold catalytic core of this domain. Thus, RNA structure and the environment of the potential methylation site are critically important for m5C RNA methylation by NSUN family methyltransferases. Notably, although the modification site motif of NSUN6 is the same for both mRNA and tRNA, namely C*UCYA (C* is the site of methylation), the recognition of tRNA and mRNA substrates by this enzyme is different and uses different combinations of the RNA-binding protein surfaces [36,37]. This suggests that m5C methyltransferases, which recognize specific structural elements in non-coding RNAs and modify cytosine residues falling into their active center, are able to modify mRNA at sites with similar but not identical structural characteristics. It appears that the subcellular localization of m5C methyltransferases, allowing them to contact mRNA, is principal for their ability to methylate mRNA at the appropriate sites.

**Figure 4:**
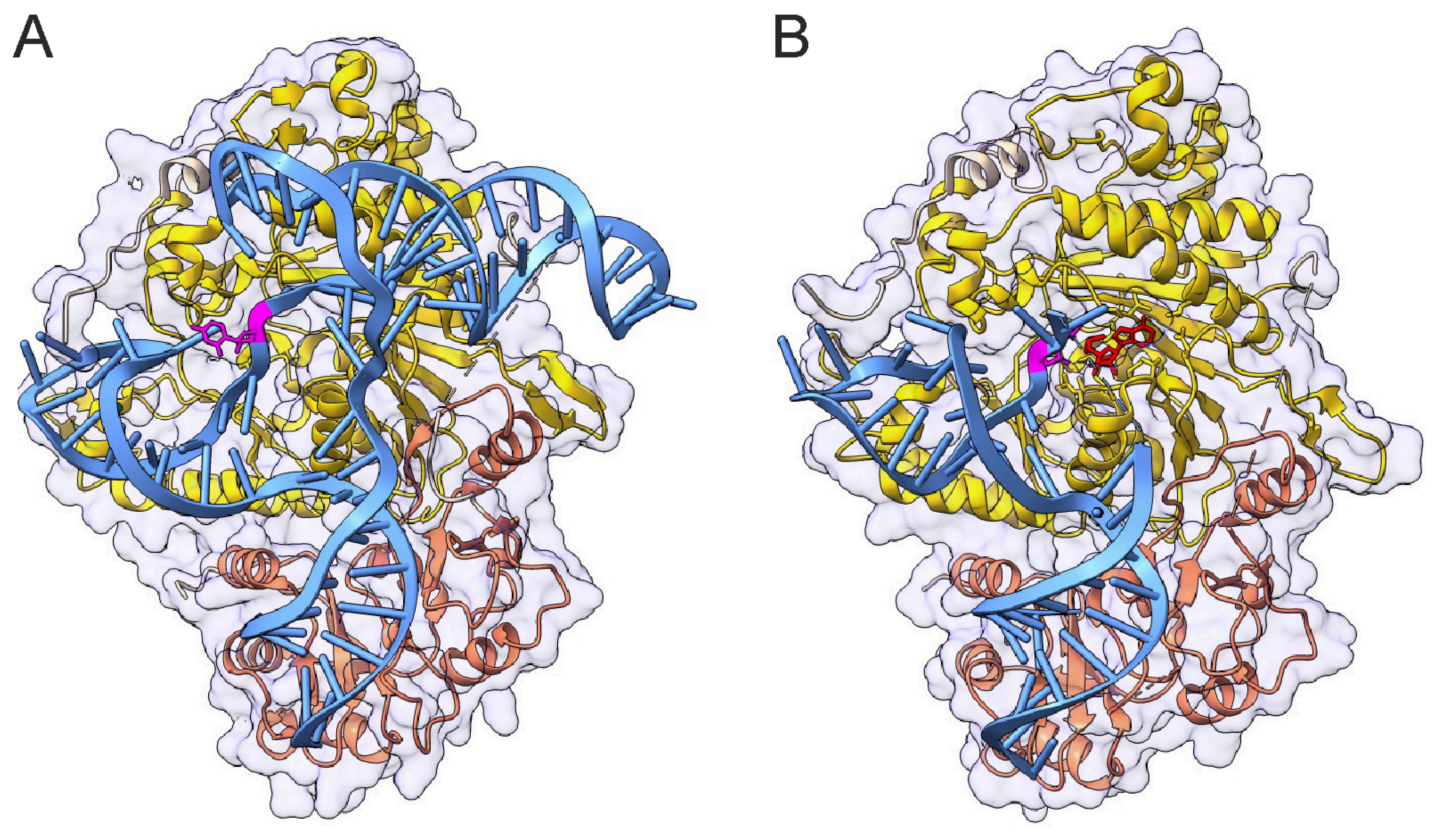
Cryo-electron microscopy structures of human NSUN2 with tRNA illustrating the interaction of the enzyme’s domains with tRNA’s arms for the C48 residue methylation. (**A**) Complex of NSUN2 with tRNA^Lys(CTT)^ (PDB: 9Z2P [42]). (**B**) Complex of NSUN2(C271A) with tRNA^Lys^ and SAM (PDB: 21ZH [43]). The color coding is the same as in Figure 3.

While the situation with the mRNA structure near the NSUN6 methylation site is more or less clear, the information on this issue for NSUN2 is somewhat contradictory. The search for a conserved motif near the methylation sites in mRNAs identified in different studies did not provide a clear result, indicating only a more frequent occurrence of G residues at positions 2–4 downstream of the C residue to be methylated [9,11,39,40,41]. This may indicate the absence of a preferential extended linear region for NSUN2 binding to mRNA near the m5C methylation site, and the binding of this enzyme to mRNA occurs due to the recognition of its fragment, which has a specific spatial structure. Only very recently, cryo-electron microscopy structures of the complex of NSUN2 with tRNA have been published [42,43] (Figure 4). The authors have found that NSUN2 recognizes various tRNA substrates based on the structure of the acceptor arm, T-arm, and variable loop, rather than on the fixed L-shaped architecture of the tRNA or any sequence motifs [43]. It has been shown that a substrate for RNA m5C methylation can indeed be any double-stem structure with a bulge junction capable of supporting stem binding at an angle, with a preference for the NNRR sequence in the stem downstream of the C residue [42]. Given the latter, one might expect a large number of mRNA methylation sites generated by NSUN2, as such structures are fairly common in long mRNAs. However, the dynamic nature of the mRNA architecture, its protection by RNA-binding proteins, and its subcellular localization, mostly different from that of NSUN2, are likely to prevent this.

## 5. mRNA m5C Readers

**Figure 5:**
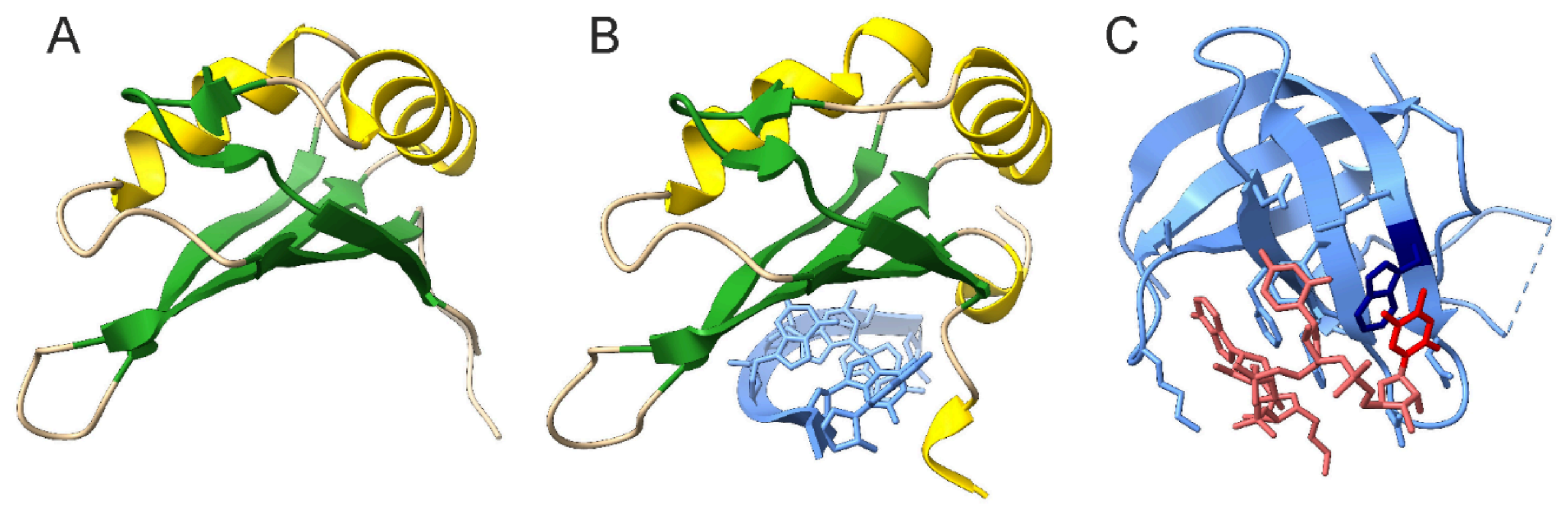
Structures of the RNA-binding domains of m5C readers. (**A**) A crystal structure of the ALYREF RRM domain showing its characteristic fold (PDB: 3ULH). Alfa-helices and beta-sheets are colored in yellow and green, respectively. (**B**) A part of the structure of the RNA-binding protein hnRNPA2B1 in complex with an RNA (blue) showing typical contacts between the RRM domain and an RNA chain (PDB: 5WWE [44]). The view and coloring are the same as in A. (**C**) Structure of the Cold Shock Domain of YBX1 (blue) in complex with m5C-modified RNA (PDB: 6A6L [45]). It is seeing a stacking between m5C (red) and a Trp residue (deep blue).

An application exploiting an RNA pull-down assay using HeLa cell nuclear extract and biotin-labeled m5C-containing 19-mer RNA to identify proteins exhibiting increased affinity for m5C sites in mRNA has revealed ALYREF/THOC4 (Aly/REF export factor/THO complex subunit 4) as the most likely candidate for the m5C reader [14]. In addition, ALYREF-immunoprecipitated mRNAs also showed several-fold higher levels of m5C modification compared to control mRNAs. Being a component of the transcription/export (TREX) complex, ALYREF participates in mRNA export and has in its structure a characteristic RNA recognition motif (RRM), which is commonly found in many RNA-binding proteins (Figure 5A,B). It appears that m5C residues (perhaps even in specific RNA contexts) promote stronger binding of RNA to the protein than non-methylated cytosine.

Another protein suggested for the role of the m5C reader was YBX1 (Y-box binding protein 1), a major RNA-binding protein in the cell, shuttled between the nucleus and cytoplasm. Similar to ALYREF, this protein has also demonstrated approximately a four-fold higher affinity for the m5C-containing RNA hexamer in HEK293T cell lysate than for the unmodified RNA [45]. Analysis of the resolved spatial structure of the YBX1 Cold Shock Domain (CSD) in complex with this RNA hexamer revealed a position of the m5C group stacked over the W65 residue (Figure 5C), suggesting a stronger hydrophobic interaction of the W65 indole ring with the methylated cytosine base than with the unmethylated one due to a hydrophobic methyl group [45]. Given the high degree of conservation of CSD in YBX1 and its homologs, one might expect that YBX1 homologs would also exhibit increased affinity for m5C sites. Indeed, it was recently shown that YBX3 CSD, like YBX1 CSD, binds more readily to a 25-mer hairpin RNA carrying m5C at the NSUN6 methylation site than to the same unmethylated hairpin [36]. Notably, the affinity of YBX3 CSD for this RNA was substantially higher than that of YBX1 CSD.

However, although the coordinated action for m5C readers and NSUN2/NSUN6 in cellular processes has been demonstrated, particularly in mRNA nucleocytoplasmic export [14] or stability [36], the mechanistic insights of this remain unclear. This is particularly relevant to how methylated mRNAs are discriminated against and how m5C readers contribute to the regulation of their biogenesis. The key issue is that the frequency of occurrence of m5C residues in mRNA is 1 in several thousand, and therefore it is unclear how the aforementioned proteins, whose affinity for m5C-containing regions in mRNA is only a few times higher than for unmodified regions, which are orders of magnitude more common, can distinguish such mRNAs.

One possible explanation could be the following: YBX1 has been shown to bind to mRNA in a cooperative manner [46,47], and an initially bound YBX1 molecule promotes multimerization of subsequent protein molecules on mRNA through the unstructured, arginine-rich C-terminal domain. Given this, even a small advantage in the binding of YBX1 to mRNA at the m5C methylation site should significantly increase the overall population of this protein on mRNA, thereby ensuring the role of YBX1 in the mRNA life (e.g., in its stability). YBX3 may act by a similar mechanism, given its high homology to YBX1. As for ALYREF, as a part of the large TREX complex [48], this small protein is unlikely to exhibit an increased specificity for m5C sites in mRNAs. However, in its free state, due to the presence of the RRM domain and N- and C-terminal disordered regions rich in arginine, such specificity would be quite expected.

## 6. mRNA m5C Erasers

In DNA, m5C epigenetic marks are removed (“erased”) by methylcytosine deoxygenases of the TET1–3 (ten-eleven translocation 1–3) family and by ALKBH1. Although some specific details of this mechanism are still subject to discussion, it is believed that these proteins are capable of stepwise oxidizing m5C in DNA via 5-hydroxymethylcytosine (hm5C) and 5-formylcytosine (f5C) up to 5-carboxylcytosine (ca5C), followed by replacement of the modified base with cytosine in the process of base excision repair (BER). All these deoxygenases have nuclear localization [49,50,51,52]; only ALKBH1 was also localized in mitochondria [25], and TET3 was detected in the cytoplasm only under specific conditions [53]. It has been noted that some of these proteins are also capable of removing m5C modification in individual RNAs (although, since complete removal of the modification does not occur in this case, it would be better to use the word “to convert”). For example, TET2 oxidizes m5C to hm5C in tRNA [54], and ALKBH1 oxidizes the residue C34 methylated by NSUN3 in mt--tRNA^Met^ to f5C [25].

If the above-mentioned m5C erasers are specific to mRNAs and are able to oxidize m5C residues within them, this likely occurs in the cell nucleus before mRNA is exported to the cytoplasm. Furthermore, C residues cannot appear at the sites of m5C because the BER mechanism is not applicable to mRNA. Moreover, recent studies show that the level of f5C, an oxidation product of m5C, in cellular mRNA is extremely low [55], as is the level of hm5C (~0.001–0.003% of total C, i.e., approximately 30–100-fold lower than the level of m5C) [56]. Thus, the process of m5C removal from mRNA, at least with the involvement of the above-mentioned proteins–erasers, is most likely a background process and is unlikely to make a significant contribution to mRNA biogenesis.

## 7. Biological Relevance of m5C Methylation of mRNA

**Figure 6:**
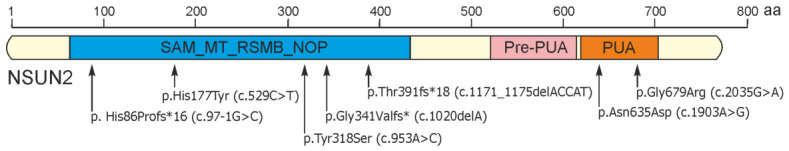
Localization of defects in the structure of NSUN2 caused by mutations in its gene, which have been described for individuals with the autosomal recessive intellectual disability. Note that the amino acid substitutions are located within the functional domains (see Figure 1) of the protein.

Deficiency or inhibition of the activity of m5C-methyltransferases specific for mRNA causes global disorders in the cellular transcriptome and translatome [18], and mutations in their genes are associated with neurodegenerative diseases and intellectual disability [57,58,59,60]. A number of mutations in the *NSUN2* gene have been described for individuals with autosomal recessive intellectual disability [57,61,62,63,64] (Figure 6). Even in *Drosophila*, a deficiency of active NSUN2 causes cognitive impairments [65]. Association of m5C methylation of mRNA with carcinogenesis has also been reported in numerous studies (e.g., [66,67,68,69]) All of this points to a critical importance of m5C-methyltransferases specific to mRNA for the life of particular mRNAs. However, it is unclear which functions of these enzymes are essential for cellular and organismal homeostasis. On the one hand, m5C methylation of tRNAs and vtRNAs is important for their maturation, and inhibition of this step should impact global cellular processes, particularly translation. On the other hand, the half-life of tRNA in cells is quite long, over 100 hours [70], meaning that it is unlikely that the mature tRNA profile in cells following NSUN2 knockdown could be reduced enough to lead to global changes in the cellular transcriptome within 24-48 hours (typical time used for the transcriptome examination in response to gene silencing in experiments with cell cultures). Given that the half-life of mRNA is much shorter, the impact of the m5C methyltransferase inactivation on the cellular transcriptome appears more probable in this respect. Furthermore, the specificity of these enzymes to individual mRNAs, rather than their impact on the translation efficiency of the entire mRNA pool, as might be the case with disrupted m5C tRNA modification, could explain the role of their deficiency in neurodegenerative and cognitive impairments. At last, the association of mutations in the NSUN2 gene with intellectual disability suggests its special importance in neurons.

Next, the level of m5C modification in mRNAs is quite low. Calculation of the number of m5C sites in mammalian mRNAs showed that the average frequency of modified cytosine residues is between 0.01 and 0.1% of the total number of cytosines, indicating that, on average, one mRNA contains a single or only a few such residues, if any. Moreover, the transcriptome analysis of f5C sites (sites formed during the sequential oxidation of the methyl group on a cytosine residue by the erasers) in mRNAs showed that their number in mRNAs is much lower than even m5C sites [55] (the number of hm5C is also substantially lower than m5C [14]). All this raises several problematic questions. Can a single small methyl group, perhaps on one (of many) cytosine residues within a long mRNA molecule, critically affect its functional properties? Is this modification essential for the biogenesis of some specific mRNAs? Is m5C mRNA methylation important only for certain cell types or only at certain stages of their differentiation? Are the positions of m5C labels in the mRNA structure/sequence important, or is their mere presence sufficient?

Given that m5C methylation of mRNA plays a functional role, it would be logical to expect different frequencies of such sites in the functional regions of mRNA (5′- and 3′-untranslated regions (UTRs) and coding sequence (CDS)), i.e., in the regions whose structure has a direct impact on its biogenesis. Meanwhile, analysis of the localization of m5C sites in mRNAs shows their ubiquitous presence in the 5′- and 3′-UTRs and CDSs, although detailed analysis demonstrates a greater tendency for m5C residues to localize in the 5′-UTR than in the 3′-UTR and their increased level in CDS near the start codon [30,71]. However, doubts remain as to whether this is due to higher cytosine levels in the 5′- UTR compared to the 3′-UTR in mRNAs. Furthermore, it has been shown that m5C methylation sites in mRNA are not evolutionarily conserved, positions of m5C modifications are generally not shared across animals, and m5C sites and containing motifs do not show greater evolutionary conservation even among mammals [72].

Recently, numerous papers have been reported on the relationship between methylation of individual mRNAs and their functioning. To a lesser extent, such works are devoted to the study of the role of NSUN6 in this process [36], and the majority of studies describe the role of NSUN2. In particular, many studies have shown that mRNAs capable of m5C methylation exhibit a reduced (usually approximately two-fold) stability in cells with NSUN2 deficiency, according to tests under conditions of transcription inhibition by actinomycin D (e.g., [73,74,75]). Overexpression of NSUN2, on the contrary, increases the stability of mRNAs capable of m5C methylation, e.g., such as mRNAs of XBP1 [76], HSBP1 [77], and MYEOV [78], measured in the same tests. And although such type studies often show only an associative relationship between the ability of specific mRNAs to m5C methylation and their level in cells with different NSUN2 expression, the role of NSUN2 in the biogenesis of specific mRNAs cannot be denied. It should also be noted that the increase/decrease in the stability of the studied m5C-methylated mRNAs is often associated with the level of m5C reader proteins, ALYREF [79,80], YBX1 [45,81,82,83], and YBX3 [36].

Despite all this, the specific mechanism by which the m5C residue in mRNA physically contributes to a decrease in its degradation remains unclear. Does m5C methylation specifically affect mRNA’s nucleocytoplasmic transport, translation, localization, or rate of degradation? It can be speculated that since ALYREF is involved in mRNA nuclear export processes, and YBX1 and YBX3 participate in mRNA biogenesis in both the nucleus and cytoplasm, mRNA methylation more actively involves m5C-methylated mRNAs in a variety of cellular events, promoting their stability. Conversely, with reduced levels of these proteins, such mRNAs become more accessible for degradation.

A link between m5C methylation of mRNA and the efficiency of its translation also remains ambiguous. It seems quite clear that the effect of enhancing/weakening mRNA translation is also related to changes in mRNA stability. With, for example, an increase in mRNA stability, it is reasonable to expect an increase in the yield of the corresponding protein product during translation, and vice versa. Methylation of the 5′- and 3′-UTR can indeed contribute to the above-described processes of mRNA involvement in cellular pathways through its interaction with ALYREF, YBX1, and other regulatory RNA-binding proteins, which may also increase mRNA stability. It can even be assumed that the observed increase in the density of m5C sites near the start codon [30,71] is somehow related to the mechanism of initiation of translation and accelerates the formation of the initiation complex, increasing ribosome occupancy on mRNA. Regarding NSUN6, its methylation sites have been shown to be predominantly located in the 3′-UTR of mRNAs in close proximity to the stop codon [39], suggesting a potential role for NSUN2 in enhancing the fidelity of translation termination on target mRNAs. The authors speculate that these m5C sites ensure termination of translation after stop codon read-through; however, they do not offer a possible molecular mechanism for this process. Meanwhile, it is unclear whether the methyl group on the cytosine residue in the mRNA CDS affects the process of decoding the A-site-bound codon on the ribosome or the movement of mRNA through the ribosome channel. The same authors [39] have obtained evidence that the m5C residue in the C*UCCA motif recognized by NSUN6 is somewhat less common in the second position of mRNA codons occupying the E- or P-site of the ribosome. Thus, it can be expected that the methyl group on the C residue does affect the translation mechanism. However, the extent of this effect and the existence of a similar effect for m5C produced by NSUN2 require clarification.

Taking all of the above into account, it can be concluded that the mRNA-specific methyltransferases NSUN2 and NSUN6 undoubtedly regulate mRNA functioning. However, whether this regulation is directly related to the m5C modification of mRNA or whether it is largely due solely to the RNA-binding properties of these methyltransferases, with mRNA methylation being merely a side effect of this binding, remains to be clarified.

## 8. Conclusions and Perspectives

Despite a significant interest in m5C methylation of mRNA and the recent progress in this area, many issues concerning this problem remain unresolved. This is largely due to a number of factors, the most important of which are as follows. First, it is required to improve the accuracy of detection of m5C sites in mRNA to rigorously exclude false signals and to obtain information about such sites in various cell types and tissues. It is possible that further advances in high-throughput nanopore sequencing, which allows for the direct detection of modified nucleotides in RNA, will solve this problem. Second, when studying the role of m5C RNA methyltransferases in cellular processes, it is important to discriminate between their functions as enzymes and as RNA-binding proteins—the components of specific ribonucleoproteins. This is necessary to understand whether m5C methylation is essential for the process under study, or whether non-catalytic functions of methyltransferase are sufficient for this when mRNA modification is a byproduct. The use of catalytically inactive methyltransferase mutants in experiments on various cell cultures, as well as expression of individual mRNAs with nucleotide substitutions at methylation sites, could clarify this issue. Third, it is important to remember that m5C RNA methyltransferases are essential participants in the processing of small RNAs, and eliminating their catalytic function will inevitably distort the results obtained with mRNA. Identifying the NSUN2 binding site on mRNA and modeling methyltransferase functions on synthetic RNA substrates in vitro could help address these issues. Without understanding the abovementioned causes, it will be difficult to answer the fundamental question of why and how a deficiency of m5C methyltransferases specific to mRNA, particularly NSUN2, leads to neuropathologies.

## Data Availability

No new data were created or analyzed in this study.
